# Internalized weight stigma and compulsive exercise among college students: a variable-centered and person-centered analysis

**DOI:** 10.3389/fpsyg.2026.1945295

**Published:** 2026-10-07

**Authors:** Xiangzi Zhou, Jianjian Bao, Haizhou Long, Dan Liang, Kanglin Wang, Fen Qiu

**Affiliations:** School of Physical Education, Wuhan University of Technology, Wuhan, China

**Keywords:** body guilt, body shame, compulsive exercise, internalized weight stigma, latent profile analysis, negative body image state

## Abstract

**Introduction:**

Internalized weight stigma may be associated with compulsive exercise through body-related self-conscious emotions and negative body image, but the underlying pathways and individual-level heterogeneity remain unclear.

**Methods:**

This cross-sectional study included 513 college students. Structural equation modeling was used to examine direct and indirect associations, and latent profile analysis was used to identify subgroups of body-related distress.

**Results:**

Internalized weight stigma was positively associated with compulsive exercise. Body shame, body guilt, and negative body image state showed significant indirect associations, including two significant serial mediation pathways. Three profiles were identified: low, moderate, and high distress. The high-distress profile had the highest compulsive exercise score.

**Discussion:**

These findings identify possible psychological pathways and a subgroup with jointly elevated distress. Because the study was cross-sectional, causal conclusions cannot be drawn.

## 1 Introduction

Physical exercise is an important means of maintaining health, regulating emotion, and supporting social adaptation among college students. However, when negative appearance evaluations and idealized body standards are repeatedly reinforced, some students may exercise primarily to control weight, modify appearance, or compensate for distress. Under these conditions, exercise may acquire compulsive, compensatory, or punitive features (Whipple et al., 2025; Reynolds et al., 2023; Smith et al., 2024). College is a developmental period in which self-concept and social identity continue to evolve, and students are particularly sensitive to peer comparison, social evaluation, and social-media content. Body anxiety, body dissatisfaction, and body image distress may therefore become more salient during this period (Edlund et al., 2022). Repeated exposure to thin and muscular ideals may lead young people to incorporate external appearance standards into their self-evaluation, reducing body acceptance and increasing the risk of maladaptive body-management behaviors (Reynolds et al., 2023; Ruiz-Turrero et al., 2022). Studies in fitness settings and among athletes have also linked exercise addiction risk to body image concerns (Corazza et al., 2019; Tariq and Saad, 2025).

Weight stigma refers to social prejudice directed at body size and weight. Its effects are not limited to external evaluation. When individuals accept and endorse negative weight-related judgments, stigma can become internalized as self-devaluation and psychological distress (Puhl et al., 2020; Romano et al., 2021). Weight stigma has also been examined in relation to exercise self-efficacy, avoidance of exercise settings, and physical activity participation (Meadows and Bombak, 2019; Pearl et al., 2021; Thedinga et al., 2021; Lucibello et al., 2023). College students who interpret judgments such as being overweight or failing to meet body ideals as evidence of personal deficiency may engage in persistent self-criticism and monitor the discrepancy between their bodies and perceived social norms. In the presence of shame, guilt, or a sense of lost control, some individuals may adopt compensatory body-management behaviors (Smith et al., 2024). From the perspective of compensatory health belief theory, exercise in this context may be driven less by health promotion than by the desire to alleviate post-eating distress, restore a sense of bodily control, or offset appearance-related evaluation pressure. Exercise may consequently become increasingly compulsive (Weinstein and Szabó, 2023). Frequent or high-intensity exercise may relieve post-eating guilt while also serving the goal of producing a socially valued appearance and reducing anticipated evaluation pressure (Vartanian and Shaprow, 2008). Accordingly, we hypothesized that internalized weight stigma would be positively associated with compulsive exercise among college students (H1).

The association between internalized weight stigma and compulsive exercise may be transmitted through body-related self-conscious emotions. In sport and clinical psychology, self-conscious emotions are viewed as a central link between external evaluation and behavioral regulation (Sabiston et al., 2021, 2022; Huellemann et al., 2021). Internalized weight stigma may be accompanied by two high-arousal negative emotions with distinct psychological drivers: body shame and body guilt. Body shame involves a global devaluation of the core self and is related to self-disgust, avoidance, and self-punishment following perceived body-related failure. It has been identified as a potential pathway linking appearance pressure to compulsive exercise in both women and men (Whipple et al., 2025; Alcaraz-Ibáñez et al., 2021). Body guilt, in contrast, focuses on the perceived failure of a specific health-management behavior and is more closely related to behavioral repair. Whether driven by self-punishment through shame or behavioral compensation through guilt, these accumulated emotions may increase the tendency to use compulsive exercise as an outlet for distress. We therefore hypothesized that body shame (H2a) and body guilt (H2b) would each mediate the association between internalized weight stigma and compulsive exercise.

The prolonged accumulation of self-conscious emotions may also lower the threshold at which body-related cues elicit distress. A body image state reflects an individual’s momentary cognitive and emotional experience of appearance and body shape in a specific context (Wang, 2012). Severe psychological distress and negative body image concerns have been associated with compulsive exercise among university students (Edlund et al., 2022). Related associations between body image concerns and problematic exercise have been reported in regular exercisers, adults, and eating-disorder samples (Gori et al., 2021a, b; Meneguzzo et al., 2022; Guo et al., 2025). We therefore hypothesized that negative body image state would mediate the association between internalized weight stigma and compulsive exercise (H3).

When internalized weight stigma co-occurs with severe shame and guilt, individuals may have fewer psychological resources with which to resist appearance-based evaluation. Compulsive exercise may then provide a temporary sense of safety or bodily compensation during periods of intense distress. Internalized weight stigma, deep self-conscious emotions, and negative body image state may thus form a linked pathway to compulsive exercise. We hypothesized that body shame or body guilt and negative body image state would form serial mediation pathways between internalized weight stigma and compulsive exercise (H4).

Existing research on the antecedents of compulsive exercise has relied largely on variable-centered methods such as structural equation modeling (SEM). These methods characterize associations among variables and average effects across a sample, but they may overlook individual differences within the college population. Objectification theory suggests that body shame, guilt, and negative body image arising under appearance pressure may not simply accumulate independently. Instead, they may interact to form distinct cognitive and emotional configurations (Fredrickson and Roberts, 1997). A single linear model cannot fully capture uneven symptom patterns or explain why only some individuals show markedly maladaptive exercise under comparable exposure to risk factors. Accordingly, the present study combined variable-centered SEM with person-centered latent profile analysis (LPA). SEM addressed the relational structure among the constructs, whereas LPA examined how these constructs co-occurred within individuals to form different risk profiles. Together, the two approaches were used to characterize the psychological association between internalized weight stigma and compulsive exercise and to identify subgroups with distinct stigma-emotion configurations.

## 2 Materials and methods

### 2.1 Participants and procedure

College students were recruited through the Wenjuanxing online survey platform. Participants completed the questionnaire anonymously. A total of 520 responses were collected. Responses were excluded when participants failed attention checks, had an extreme completion time, or showed patterned responding. The final sample comprised 513 valid responses. The sample included 288 women (56.14%) and 225 men (43.86%). Participants were 18–23 years old (*M* = 20.46, SD = 1.74). Height ranged from 150 to 191 cm (*M* = 169.49, SD = 7.77), weight ranged from 40 to 95 kg (*M* = 60.96, SD = 11.34), and BMI ranged from 13.21 to 30.67 kg/m^2^ (*M* = 21.13, SD = 3.11). According to Chinese adult BMI criteria, 101 participants were underweight (19.69%), 314 had a normal weight (61.21%), 88 were overweight (17.15%), and 10 met criteria for obesity (1.95%).

The study received approval from the relevant ethics committee. All participants volunteered to take part and reviewed and confirmed informed consent before completing the questionnaire.

### 2.2 Measures

#### 2.2.1 Negative body image state

Negative body image state was assessed with the Body Image States Scale validated in a Chinese college-student sample (Wang, 2012). The scale assesses immediate cognitive and emotional experiences related to body shape, weight, and physical attractiveness in specific contexts. Items were rated on a 9-point Likert scale and reverse-scored where appropriate, with higher scores indicating more severe negative body image state. Cronbach’s alpha was 0.890 in the present sample. The one-factor measurement model showed good fit, χ^2^/df = 1.21, RMSEA = 0.020, CFI = 0.998, TLI = 0.996, and SRMR = 0.011. Standardized factor loadings ranged from 0.77 to 0.86.

#### 2.2.2 Body shame and body guilt

Body-related self-conscious emotions were assessed with the Body Shame and Body Guilt Scale used in Chinese college-student research (Bi, 2017). The scenario-based measure assesses shame and guilt in response to body-related negative events. For the present study, the body shame and body guilt dimensions were retained, whereas the externalized-blame dimension was excluded because it is directed toward external causes rather than self-directed emotion. The retained items were rated on a 5-point Likert scale, with higher scores indicating greater body shame or body guilt. Confirmatory factor analysis supported the revised two-factor model, χ^2^/df = 1.23, RMSEA = 0.021, CFI = 0.992, TLI = 0.991, and SRMR = 0.027. Cronbach’s alpha coefficients were 0.940 for body shame, 0.946 for body guilt, and 0.927 for the total retained scale.

#### 2.2.3 Internalized weight stigma

Internalized weight stigma was measured with the Internalized Weight Stigma Scale developed by Lillis et al. (2010). The scale assesses the extent to which weight stigma is internalized as self-devaluation and fear of weight stigma. Items were rated on a 5-point Likert scale, with higher scores indicating greater internalized weight stigma. Cronbach’s alpha was 0.920 in the present sample. The one-factor model showed good fit, χ^2^/df = 1.89, RMSEA = 0.042, CFI = 0.976, TLI = 0.968, and SRMR = 0.029. Standardized factor loadings ranged from 0.69 to 0.81.

#### 2.2.4 Compulsive exercise

Compulsive exercise was assessed with the Compulsive Exercise Test (CET) developed by Taranis et al. (2011). The measure covers avoidance and emotion regulation, weight-control exercise, mood improvement, lack of exercise enjoyment, and exercise rigidity. Total or mean scores were used to represent the severity of compulsive exercise, with higher scores indicating stronger compulsive exercise tendencies. Cronbach’s alpha coefficients for the five dimensions were 0.88, 0.90, 0.94, 0.82, and 0.81, respectively, and the coefficient for the total scale was 0.970.

### 2.3 Analytical strategy

Analyses were conducted at both the variable and person levels. SEM was used to estimate the direct and indirect associations among internalized weight stigma, body shame, body guilt, negative body image state, and compulsive exercise. Item parceling was used to reduce model complexity after confirmatory factor analyses supported the unidimensionality of the relevant dimensions. Indirect effects were tested with 5,000 bootstrap resamples and 95% confidence intervals. LPA used the mean scores of internalized weight stigma, body shame, body guilt, and negative body image state as indicators (Wang and Bi, 2018). The number of profiles was selected using AIC, BIC, adjusted BIC, the Lo–Mendell–Rubin likelihood ratio test, the bootstrap likelihood ratio test, profile interpretability, and parsimony (Nylund et al., 2007). BCH tests were then used to compare compulsive exercise across the latent profiles.

## 3 Results

### 3.1 Common method bias

Harman’s single-factor test was used as a preliminary assessment of common method bias. An unrotated exploratory factor analysis of all scale items extracted multiple common factors with eigenvalues greater than 1. The first factor explained 36.074% of the variance, below the conventional 40% threshold, suggesting that common method bias was unlikely to pose a serious threat in this dataset.

### 3.2 Descriptive statistics and correlations

Descriptive statistics and correlations are presented in Table 1. Compulsive exercise showed its strongest correlation with negative body image state (*r* = 0.518, *p* < 0.001), followed by its correlations with internalized weight stigma (*r* = 0.479, *p* < 0.001) and the correlation between negative body image state and internalized weight stigma (*r* = 0.462, *p* < 0.001). Body guilt and body shame showed the smallest correlation in the matrix (*r* = 0.166, *p* < 0.001), although the association remained statistically significant.

**Table 1:** Descriptive statistics and intercorrelations among body guilt, body shame, compulsive exercise, negative body image state, and internalized weight stigma (*N* = 513).

Variable	*M*	SD	1	2	3	4
BG	39.06	11.53	1			
BS	38.89	11.57	0.166***	1		
CET	83.89	27.42	0.406***	0.432***	1	
NBI	24.84	9.13	0.369***	0.388***	0.518***	1
IWS	27.08	7.95	0.282***	0.404***	0.479***	0.462***

BG, body guilt; BS, body shame; CET, compulsive exercise; NBI, negative body image state; IWS, internalized weight stigma. *p < 0.05, **p < 0.01, ***p < 0.001.

### 3.3 Mediation analysis

#### 3.3.1 Item parceling and model fit

The core variables contained a relatively large number of observed items. To control item-level measurement error and improve the ratio of indicators to estimated parameters, item parceling was conducted following the recommendations of Wu and Wen (2011). Unidimensionality was confirmed with confirmatory factor analysis before parceling. Using a balancing approach, items were ordered by factor loading, and the highest- and lowest-loading items were combined to form the first parcel, followed by the next highest- and next lowest-loading items. This process was repeated until the parcels were formed. The 13 body guilt items were combined into four observed parcels (BG_P1–BG_P4), and the 13 body shame items were combined into four observed parcels (BS_P1–BS_P4). Internalized weight stigma was represented by three parcels, negative body image state by two parcels, and compulsive exercise by six parcels. Mean parcel scores were entered as observed indicators of the corresponding latent variables.

The SEM showed good overall fit, χ^2^ = 142.315, df = 142, χ^2^/df = 1.002, GFI = 0.972, AGFI = 0.962, RMR = 0.019, RMSEA = 0.002, NFI = 0.987, RFI = 0.984, CFI = 0.977, TLI = 0.954, and SRMR = 0.029. All indices met commonly used criteria in psychological research. The very low RMSEA should be interpreted cautiously because item parceling can reduce indicator-level random error and may produce an RMSEA estimate close to zero when the sample size is moderate and the number of parcels is small. The other fit indices and the factor loadings provided convergent evidence of adequate model-data fit. Parcel loadings ranged from 0.788 to 0.941, and all were significant at *p* < 0.001 (Figure 1).

**Figure 1:**
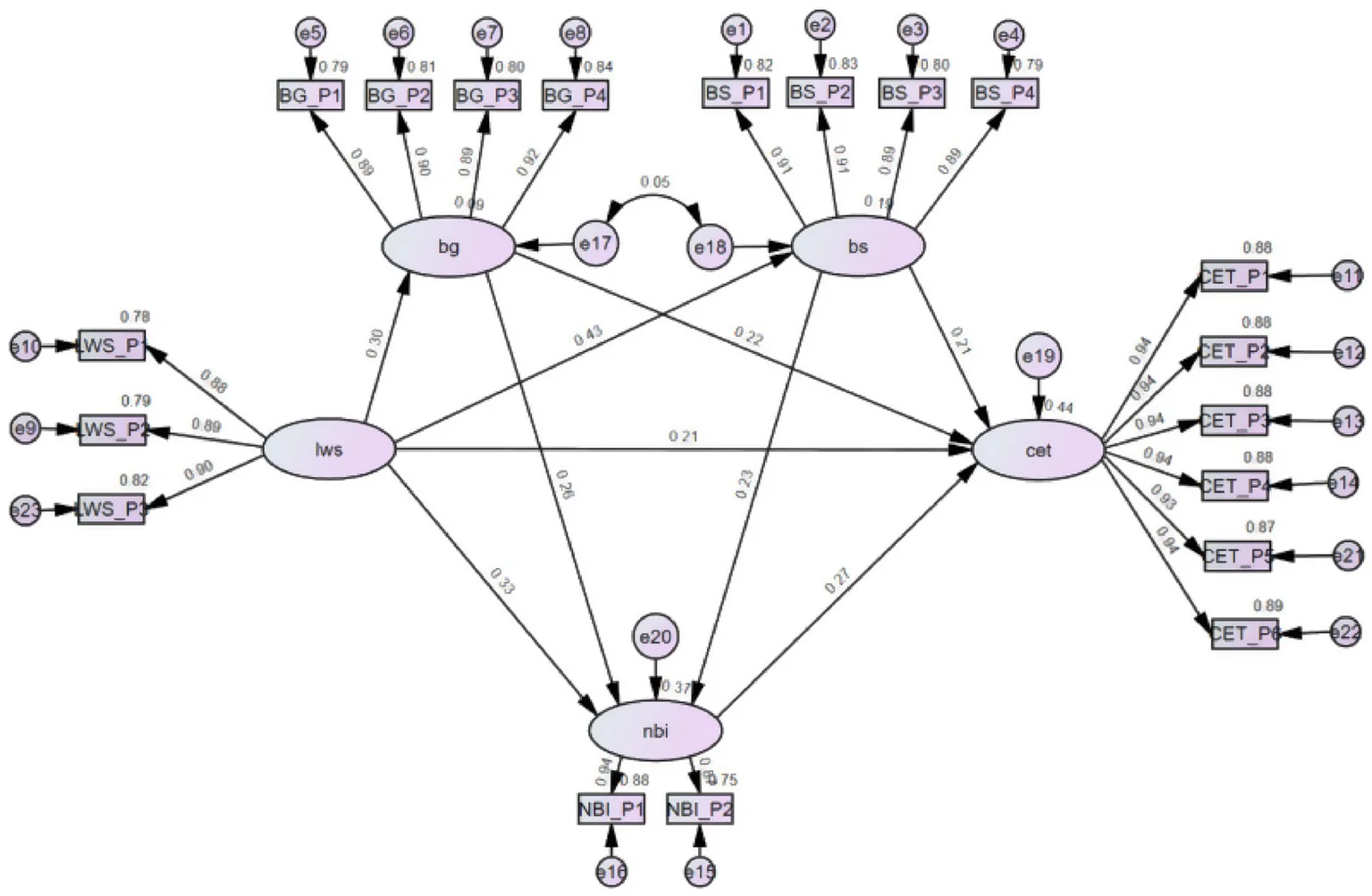
Structural model linking internalized weight stigma, body shame, body guilt, negative body image state, and compulsive exercise.

#### 3.3.2 Indirect effects

Bootstrap analysis with 5,000 resamples and 95% confidence intervals was used to test indirect effects. The serial indirect effect through internalized weight stigma → body guilt → negative body image state → compulsive exercise was 0.028, with a 95% confidence interval of [0.016, 0.047]. The corresponding serial indirect effect through body shame was 0.036, with a 95% confidence interval of [0.020, 0.062]. Neither interval included zero. The specific indirect effects through body guilt (0.089, 95% CI [0.052, 0.137]), body shame (0.124, 95% CI [0.073, 0.183]), and negative body image state (0.120, 95% CI [0.075, 0.184]) were also significant. The direct effect was 0.289, and the total indirect effect was 0.397, yielding a total effect of 0.687. Detailed path estimates are presented in Table 2.

**Table 2:** Path analysis of the mediation model linking internalized weight stigma, body shame, body guilt, negative body image state, and compulsive exercise.

Effect	Path	Estimate	95% CI	Proportion of total effect (%)
Direct effect	IWS → CET	0.289	[0.167, 0.411]	42.07
Indirect effect	IWS → BG → CET	0.089	[0.052, 0.137]	12.95
	IWS → BS → CET	0.124	[0.073, 0.183]	18.05
	IWS → NBI → CET	0.120	[0.075, 0.184]	17.47
	IWS → BG → NBI → CET	0.028	[0.016, 0.047]	4.08
	IWS → BS → NBI → CET	0.036	[0.020, 0.062]	5.24
Total indirect effect		0.397	[0.317, 0.486]	57.79
Total effect		0.687	[0.584, 0.790]	100.00

### 3.4 Latent profiles of body-related distress

LPA was conducted to examine heterogeneity in body-related risk factors. The mean scores of internalized weight stigma, body shame, body guilt, and negative body image state were used as indicators. As the number of profiles increased, AIC, BIC, and adjusted BIC decreased. Although the four-profile solution had lower information criteria, its LMR test was no longer significant (*p* = 0.1187). Considering model parsimony, profile proportions, and theoretical interpretability, the three-profile solution was retained (Table 3). Its entropy was 0.629, below the ideal value of 0.80, indicating that the profile boundaries were not fully distinct.

**Table 3:** Fit indices for latent profile models with different numbers of classes.

Classes	AIC	BIC	aBIC	Entropy	LMR (p)	BLRT (p)	Class proportions (%)
2	5794.221	5849.344	5808.080	0.673	< 0.001	< 0.001	54.2/45.8
3	5760.916	5837.241	5780.106	0.629	0.0395	< 0.001	53.6/18.9/27.5
4	5740.699	5838.225	5765.220	0.635	0.1187	< 0.001	23.2/28.3/28.3/20.3

The three profiles were labeled low distress, moderate distress, and high distress based on their scores across the four indicators. Average posterior probabilities were 0.807, 0.815, and 0.872, respectively, indicating acceptable classification accuracy despite the modest entropy (Table 4). The low-distress profile (*n* = 97, 18.91%) had the lowest scores on all four indicators and represented students with little evidence of internalized weight stigma and generally adaptive body-related emotional experiences. The moderate-distress profile (*n* = 275, 53.61%) showed scores close to the sample average, representing the majority of students who were attentive to body weight and shape but did not show pronounced distress. The high-distress profile (*n* = 141, 27.48%) showed jointly elevated internalized weight stigma, body shame, body guilt, and negative body image state. This coordinated elevation suggests that internalized stigma, deeper self-conscious emotions, and momentary body image distress may co-occur rather than operate as isolated features, consistent with the cascading logic of objectification theory (Fredrickson and Roberts, 1997) (Figure 2).

**Figure 2:**
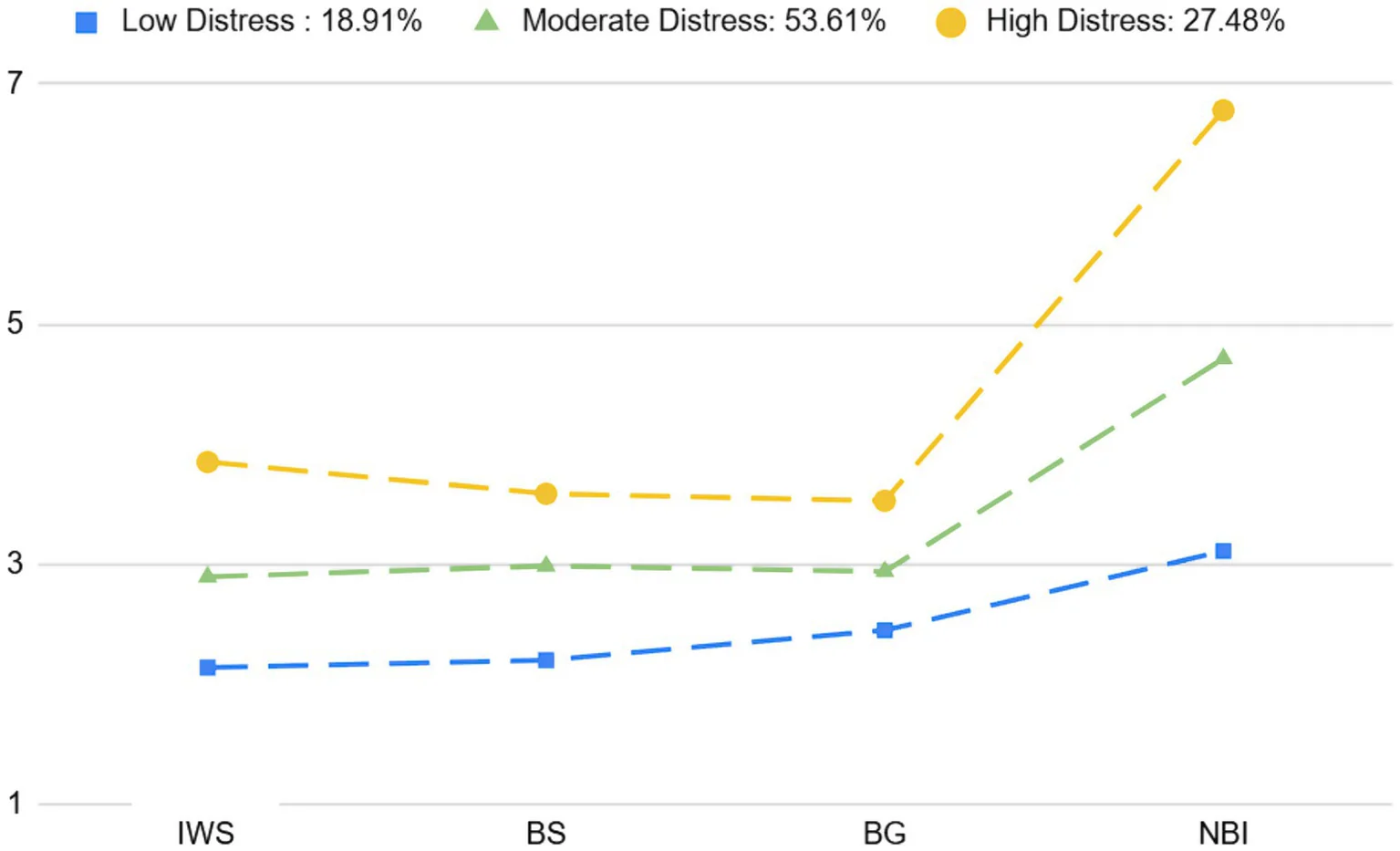
Latent profiles based on internalized weight stigma, body shame, body guilt, and negative body image state.

**Table 4:** Classification quality for the three-profile solution.

Assigned profile	Low distress	Moderate distress	High distress
Low distress	0.807	0.105	0.087
Moderate distress	0.184	0.815	0
High distress	0	0.128	0.872

Diagonal values are average posterior probabilities; off-diagonal values represent classification error.

To examine external validity, BCH tests compared the profiles on compulsive exercise. The overall difference was significant, χ^2^ = 269.158, *p* < 0.001. The low-distress profile had a mean CET score of 2.20, the moderate-distress profile had a mean score of 3.44, and the high-distress profile had a mean score of 4.55. Thus, compulsive exercise increased across the three profiles in parallel with body-related distress. Approximately 27.5% of participants belonged to the high-distress profile, indicating that a substantial subgroup of college students may experience jointly elevated stigma internalization and body-related emotional distress together with stronger compulsive exercise tendencies (Table 5).

**Table 5:** Means, standard deviations, and proportions of indicators across latent profiles.

Indicator	Low distress	Moderate distress	High distress
*n*	97	275	141
Proportion (%)	18.91	53.61	27.48
IWS	2.14 ± 0.13	2.90 ± 0.09	3.85 ± 0.08
BS	2.20 ± 0.13	2.99 ± 0.12	3.59 ± 0.07
BG	2.45 ± 0.12	2.94 ± 0.08	3.53 ± 0.09
NBI	3.11 ± 0.43	4.71 ± 0.17	6.78 ± 0.22
CET	2.20 ± 0.12	3.44 ± 0.08	4.55 ± 0.09

## 4 Discussion

### 4.1 Direct association between internalized weight stigma and compulsive exercise

After body shame, body guilt, and negative body image state were included as mediators, the direct association between internalized weight stigma and compulsive exercise remained significant and accounted for 42.07% of the total effect. The direct and emotional pathways therefore jointly characterized the association. This pattern is consistent with the possibility that compulsive exercise is maintained both by immediate relief from negative emotion and by a more stable motivation to compensate for perceived body-related deficiency. The direct pathway also indicates that the three emotional mediators examined here do not fully explain the association. Future studies should examine additional processes, such as habitual exercise strength, exercise self-efficacy, and reinforcement from appearance-based goals.

### 4.2 Mediating roles of body shame, body guilt, and negative body image state

Internalized weight stigma was associated with compulsive exercise through three specific indirect pathways and two serial pathways. The total indirect effect accounted for 57.79% of the total effect, suggesting that body-related self-conscious emotions and negative body image state may carry a substantial part of the association. The body shame pathway (0.124, 18.05%) and negative body image pathway (0.120, 17.47%) were similar in magnitude, whereas the body guilt pathway (0.089, 12.95%) was smaller. This pattern corresponds to the conceptual distinction between shame and guilt. Shame involves a global negative evaluation of the self and may promote avoidance or self-punishment, whereas guilt focuses on a specific behavior and may promote behavioral repair (Sabiston et al., 2021). The stronger association between shame and compulsive exercise may reflect the stable self-devaluing cognition that can maintain a rigid, emotion-regulation-dependent behavior pattern.

Negative body image state showed two roles in the model. It operated as an independent mediator that connected internalized weight stigma to a momentary body image crisis, and it functioned as the final mediator in both serial pathways. This structure is consistent with objectification theory. Prolonged exposure to an evaluative gaze may first undermine deeper self-evaluation, then lower the threshold for negative reactions to everyday body-related cues. The resulting momentary image distress may be closely proximal to the initiation of compulsive exercise. Although the serial indirect effects were statistically significant, their absolute magnitudes were small, so their practical and clinical significance should be interpreted cautiously.

The two serial pathways shared the same terminal structure: internalized weight stigma → body shame or body guilt → negative body image state → compulsive exercise. This pattern offers a testable temporal hypothesis for future longitudinal or ecological momentary assessment studies. Internalized stigma may contribute to deeper self-conscious emotions, which may intensify context-specific body image distress before compulsive exercise is initiated. The present cross-sectional data do not establish this temporal sequence, but they identify a plausible configuration for further testing.

### 4.3 Person-level heterogeneity and implications

The LPA identified three heterogeneous profiles based on internalized weight stigma, body shame, body guilt, and negative body image state: low distress (18.91%), moderate distress (53.61%), and high distress (27.48%). The high-distress profile showed a coordinated elevation across all four indicators rather than a pattern in which internalized stigma was high but emotion remained low, or emotion was high despite limited internalization. The alignment between the SEM and LPA results is informative. SEM identified multiple pathways from internalized stigma through body shame, body guilt, and negative body image state to compulsive exercise, whereas LPA identified a subgroup in which the same risk indicators co-occurred and compulsive exercise was highest. The two analytic perspectives therefore support a common conclusion while addressing different questions: SEM describes how variables are related, and LPA describes how these variables are organized within individuals.

The high-distress profile accounted for more than one quarter of the sample. This proportion should not be interpreted as a clinical prevalence estimate, because the study used a nonclinical convenience sample and the profile solution had modest entropy. Nevertheless, the profile may identify a subgroup that is easily missed by general screening focused mainly on depression or anxiety. Screening and intervention should therefore consider body image distress, internalized weight stigma, and maladaptive exercise tendencies together. For students in the low-distress profile, universal education emphasizing body functionality and health-oriented exercise may help maintain adaptive adjustment. For students in the moderate-distress profile, prevention should focus on preventing ordinary body concern from escalating into persistent distress. For students in the high-distress profile, coordinated support from counseling services and physical education programs may be needed, with attention to body image reappraisal, differentiation of shame and guilt, and a shift from appearance correction toward self-care and functional goals. Evidence from eating-disorder treatment settings also highlights the potential value of exercise psychoeducation for addressing compulsive exercise (Hallward et al., 2022).

### 4.4 Limitations

Several limitations should be considered. First, the cross-sectional design does not establish temporal ordering or causality. Longitudinal, experimental, ecological momentary assessment, or experience-sampling designs are needed to examine dynamic relations among weight stigma experiences, body shame and guilt, negative body image state, and compulsive exercise urges. Second, participants were recruited through an online convenience sample, and the study did not stratify recruitment by region, institution type, or academic major. This may limit generalizability to the broader college population. Stratified or cluster sampling would provide a stronger test of replication. Third, the entropy of the three-profile solution was below the ideal threshold, so profile boundaries should be interpreted cautiously and replicated in larger independent samples. Future studies could also include body appreciation, self-compassion, muscle dysmorphia, BMI or weight status, and exercise frequency to distinguish risk, buffering, and adaptive body-related profiles more precisely.

## 5 Conclusion

Internalized weight stigma was directly and indirectly associated with compulsive exercise among college students. Body shame, body guilt, and negative body image state were significant psychological pathways, including two serial mediation pathways. LPA further identified three subgroups, and the high-distress profile had the highest compulsive exercise score. These findings support the value of integrating variable-centered and person-centered approaches when studying body-related risk and compulsive exercise. Because the present study was cross-sectional, the findings provide an empirical basis for screening and intervention priorities rather than evidence of a causal intervention model.

Universities may consider including brief assessments of body image distress, internalized weight stigma, and maladaptive exercise tendencies in relevant health education or mental health screening, while recognizing that their predictive validity requires longitudinal evaluation. Counseling services may explore group interventions focused on body image reappraisal and self-acceptance, and physical education programs may emphasize bodily capability, exercise experience, and health benefits rather than appearance-based outcomes. The effectiveness of these approaches should be tested in controlled intervention studies.

## Data Availability

The original contributions presented in the study are included in the article/supplementary material, further inquiries can be directed to the corresponding author.
